# Factors associated with a history of critical wandering among Medic-Alert subscribers

**DOI:** 10.1186/s12877-024-05162-3

**Published:** 2024-06-28

**Authors:** Antonio Miguel Cruz, Hector Perez, Emily Rutledge, Christine Daum, Lili Liu

**Affiliations:** 1 Department of Occupational Therapy. Faculty of Rehabilitation Medicine, University of Alberta, 2-64 Corbett Hall, Edmonton, AB T6G 2G4 Canada; 2 Innovation & Technology (GRRIT), Glenrose Rehabilitation Research, Glenrose Rehabilitation Hospital, 10230 111 Ave NW, Edmonton, AB T5G 0B7 Canada; 3 School of Public Health Sciences, Faculty of Health, University of Waterloo, 200 University Ave W, Waterloo, ON N2L 3G5 Canada

**Keywords:** Cognitive impairment, Alzheimer's disease, Missing incident, Classification, Logistics regression

## Abstract

**Background:**

Critical wandering occurs when an individual living with dementia leaves a location and is unaware of place or time. Critical wandering incidents are expected to increase with the growing prevalence of persons living with dementia worldwide. We investigated the association between demographic, psychopathological, and environmental factors and a history of critical wandering among Medic-Alert subscribers, both with and without dementia.

**Methods:**

Our retrospective study included data of 25,785 Canadian Medic-Alert subscribers who were aged 40 years or older. We used multivariable logistic regression analysis to examine the associations between a history of critical wandering and dementia status as psychopathological independent variable, controlled by demographic (age, ethnic background, sex at birth, Canadian languages spoken) and environmental (living arrangement, population density) factors.

**Results:**

The overall study sample comprised of mainly older adults (77.4%). Medic-Alert subscribers who were older, male sex at birth, living with dementia, of a minority ethnic group and who did not have proficiency in an official Canadian language had a higher likelihood of a history of critical wandering. Residing in an urban environment, in an institution or with a family member, were environmental factors associated with a higher likelihood of a history of critical wandering.

**Conclusions:**

People living with dementia experience a higher likelihood of a history of critical wandering compared to those without dementia. Medic-Alert and similar organizations can develop algorithms based on the associated factors that can be used to flag risks of critical wandering. This can inform preventative strategies at the individual and community levels.

**Supplementary Information:**

The online version contains supplementary material available at 10.1186/s12877-024-05162-3.

## Background

Dementia is a serious public health issue. Roughly 5% of the world’s older population live with dementia [1, 2]. It is estimated that globally nearly 10 million people are diagnosed with dementia every year, an incidence statistic that translates into one new case every three seconds [2]. At this rate, people with dementia are predicted to reach 132 million by 2050 [2]. In Canada, at least 600,000 people currently are living with dementia [3]. By 2030, the number of Canadians with dementia will reach at least 1,712,400, roughly a 4.5% of the Canadian population [3].

The disease burden cost associated with dementia is sizable. In 2015, worldwide dementia costs were equivalent to 1.1% of global gross domestic product [2]. The annual cost of dementia to the Canadian economy and healthcare system is over $10.4 billion and it is expected to double by 2031 [4]. As dementia leads to increased costs for governments, communities, families and people living with dementia, policies, strategies, plans for dementia needs to be in place to reduce the risks associated to dementia [2].

With increasing numbers of people living with dementia, the prevalence of critical wandering is rising as well. Critical wandering refers to when an individual living with dementia “leaves an institution or home [with or without knowledge of their care partner] and is unaware of his or her situation in terms of place and/or time” [5]. People with dementia who face behavioural changes such as critical wandering experience increased risks of getting lost [6, 7]. If a person with dementia gets lost and is not found relatively quickly, they might experience serious injury or even death [8]. This is especially true in locations with inclement weather. Thus, comprehensive assessments and tools for persons who are at risk of getting lost due to critical wandering are paramount.

Validated risk models to identify older adults living with dementia at high risk of getting lost due to critical wandering do not exist [9]. There is evidence that demographic (e.g., age, sex at birth) [10], psychopathological (e.g., mental disabilities/cognitive capacity, i.e., Alzheimer’s dementia or other types) [6], and environmental and situational (e.g., economic state, access to healthcare services) [9, 11] are factors associated to getting lost in this population [9]. However, prior studies have had significant limitations. Retrospective studies are limited by small sample sizes, poor data quality, and missing data [9, 12–14], leading to a limited scope of the statistical analyses.

To address this knowledge gap, this study aimed to investigate the association between demographic, psychopathological, and environmental factors and a history of critical wandering among Medic-Alert subscribers, both with and without dementia. We used records from Medic-Alert Foundation Canada (*n* = 25,785) to provide estimates of the factors associated with a history of critical wandering among individuals living with and without dementia.

### Theoretical framework

People living with dementia are at risk of unintentionally getting lost due to critical wandering. A missing incident in people living with dementia can occur when a person is left unsupervised for a few minutes [15]. According to Ferguson [9], there are factors that explain the phenomenon of missingness in persons living with and without dementia. These factors can be classified as true risk factors (also known as antecedents), or correlating factors (also known as consequences, concomitants, or correlates). To be considered a true risk factor, a factor must involve measurable characteristics of each subject in a specified population that precede the outcome of interest.

It has been reported that factors, whether antecedents or correlates, encompass demographic, psychopathological, environmental, and situational elements [9]. In this study, demographic factors of particular interest include personal characteristics (e.g., age, sex at birth, gender, ethnicity). Psychopathological factors consider manifestation of behaviours related to cognitive or psychological impairment, mental illness, disorders or distress. In this domain, characteristics of interest include whether or not the person is living with dementia. The several neurocognitive deficits that arise with the progression of dementia predisposes people to an inability to safely wayfind [16]. These deficits include remembering facts and events, short term memory for recent events and contexts, anosognosia (or impaired insight), executive function impairments, and changes to visuospatial and visuoperceptual processing (difficulty with navigation). Finally, environmental and situational factor, which include characteristics outside of a person (e.g., social, cultural, political, economic, weather conditions), can impact missingness. For example, lack of access due to affordable respite care, medications, or dementia-specific services increases the potential for a missing incident as it is more likely a person is left unsupervised [17]. In the environmental and situational factor, characteristics of particular interest in this study include population density and living arrangements.

## Methods

### Study design and sample

In this cross-sectional retrospective quantitative study, the target population comprised 25,785 Medic-Alert subscribers aged 40 years and older, including individuals both with and without dementia. Data were collected from January 2015 to July 2021.

### Ethics

The need for informed consent was waived by the University of Waterloo ethics review board, because of the retrospective nature of the study involving deidentified data. Ethics clearance was obtained through the University of Waterloo Office of Research Ethics (Study code protocol: 43164).

### Variables

#### Outcome variable

The outcome measure of interest in this study was a history of critical wandering. Critical wandering was defined as a behavior that occurs when a person leaves home or a facility unaccompanied and is unaware of their location or time [18]. Data on the history of critical wandering were self-reported by Medic-Alert subscribers. Specifically, Medic-Alert subscribers were asked whether they had a history of critical wandering, with response options including “never,” “1–4 times,” or “more than 4 times”.

### Independent variables

Table 2 in the supplemental material shows the domains, categories, and operationalization of the independent variables. The independent variables of interest include demographic, psychopathological, and environmental factors, as well as situational domains.

#### Age (in number of years)

Refers to the chronological age of an individual in years. The age variable was grouped into six broader categories. Data on age were self-reported by the Medic-Alert subscribers.

#### Sex at birth

Refers to sex assigned at birth. This variable may also be understood as the sex recorded at a person's birth. Categories used for this variable were male and female. Data on sex at birth was self-reported by the Medic-Alert subscribers.

#### Ethnic background

Refers to the ethnic or cultural origins of the person's ancestors. The ethnic background was combined into four broader categories, i.e., White (Caucasian), Chinese, Black, and Others (see Table 1 note (b) for more details). Ethnic background data was self-reported by the Medic-Alert subscribers.

**Table 1:** Demographic, psychopathological, and environmental variables and prevalence of no history of wandering versus history of wandering incidents among Medic-Alert subscribers (*n* = 25,785)

Variables ^a^	Critical Wandering		Total	Statistical Tests (No critical wandering vs. critical wandering)
	**No** **n (%)** **18,436 (71.5)**	**Yes** **n (%)** **7,349 (28.5)**	**n** **(%)** **25,785 (100)**	
**Age group**				
≤ 65	5,499 (29.8)	328 (4.5)	5,827 (22.6)	χ^2^(5) = 2682.50, *p *< 0.000
65–74	3,450 (18.7)	846 (11.5)	4,296 (16.7)	
75–84	4,855 (26.3)	2,699 (36.7)	7,554 (29.3)	
85–94	4,052 (22.1)	2,984 (40.6)	7,036 (27.3)	
95–104	574 (3.1)	482 (6.6)	1,056 (4.1)	
≥ 105	6 (0.03)	10 (0.14)	16 (0.1)	
**Sex at birth**				
Female	10,118 (54.9)	3,845 (52.3)	13,963 (54.2)	χ^2^(1) = 13.89, *p* < 0.001
Male	8,318 (45.1)	3,504 (47.7)	11,822 (45.8)	
**Ethnic background**				
White (Caucasian)	16,584 (90.0)	6,170 (84.0)	22,754 (88.2)	χ^2^(3) = 196.50, *p* < .001
Other ^b^	1,225 (6.6)	717 (9.8)	1,942 (7.5)	
Chinese	359 (1.9)	239 (3.3)	598 (2.3)	
Black ^c^	268 (1.5)	223 (3.0)	491 (1.9)	
**Canadian languages spoken **^**d**^				
Yes	18,023 (97.8)	6,892 (93.8)	24,915 (96.6)	χ^2^(1) = 255.087, *p* < 0.001
No	413 (2.2)	457 (6.2)	870 (3.4)	
**Province/Territory**				
Ontario	11,220 (60.9)	4,747 (64.6)	15,967 (61.9)	χ^2^(11) = 159.72, *p* < 0.001
Quebec	2,161 (11.7)	993 (13.5)	3,154 (12.2)	
British Columbia	2,036 (11.0)	813 (11.1)	2,849 (11.0)	
Alberta	1,251 (6.8)	319 (4.3)	1,570 (6.1)	
Manitoba	602 (3.3)	213 (2.9)	815 (3.2)	
Nova Scotia	442 (2.4)	108 (1.5)	550 (2.1)	
Saskatchewan	309 (1.7)	89 (1.2)	398 (1.5)	
New Brunswick	226 (1.2)	37 (0.5)	263 (1.0)	
Newfoundland and Labrador	107 (0.6)	18 (0.2)	125 (0.5)	
Prince Edward Island	58 (0.3)	7 (0.1)	65 (0.3)	
Yukon	13 (0.07)	5 (0.07)	18 (0.07)	
Nunavut/Northwest Territories	10 (0.05)	1 (0.01)	11(0.042)	
**Population Density**				
Urban	16,344 (88.7)	6,841 (93.1)	23,185 (89.9)	χ^2^(1) = 113.98, *p* < 0.001
Rural	2,092 (11.3)	508 (6.9)	2,600 (10.1)	
**Living arrangement**				
With Family	12,342 (66.9)	4,456 (60.6)	16,798 (65.1)	χ^2^(3) = 639.430, *p* < 0.001
Alone	3,440 (18.7)	1,073 (14.6)	4,513 (17.5)	
Institution ^e^	1,937 (10.5)	1,639 (22.3)	3,576 (13.9)	
Other ^f^	717 (3.9)	181 (2.5)	898 (3.5)	
**Dementia status**				
Present	7,503 (40.7)	5,561 (75.7)	13,064 (50.7)	χ^2^(1) = 2571.11, *p* = 0.000
Not present	10,933 (59.3)	1,788 (24.3)	12,721 (49.3)	

^a^Variable domains/factors: Demographi**c**: Age group, Ethnic background, Sex at birth, Canadian languages spoken. Environmental: Living arrangement, Population Density. Psychopathological: Dementia status

^b^Arab/West Asian (e.g., Armenian, Egyptian, Iranian), Latin American, South Asian, Korean, Mediterranean, first nations (e.g., Inuit, Métis, North American Indian), Filipino, Caribbean/West Indian (Lucia, Antigua), Southeast Asian, and Japanese

^c^African. Haitian. Jamaican. Somali

^d^English or French

^e^Special care facilities, such as nursing homes, chronic care and long-term care hospitals, and residences for senior citizens, Assisted Living, and Continuous Care Communities

Co-living arrangements, shared housing, living with roommates, or unconventional living situations like living on a boat or in a tiny house

#### Canadian languages spoken

Refers to whether the person can conduct a conversation in the official languages of Canada, i.e., English only, French only, in both or in neither language. This variable was combined into two broader categories, i.e., Yes or No. Knowledge of the official languages of Canada data was self-reported by the Medic-Alert subscribers.

#### Province/Territory

This variable refers to the province or territory of residence of the Medic-Alert subscriber. In Canada, provinces and territories are the primary political divisions. Therefore, our study utilized the 13 categories corresponding to Canada's division, comprising of 10 provinces and 3 territories. Due to the limited number of Medic-Alert subscribers from Nunavut and the Northwest Territories, we have combined them into a single category. Province/territory data were self-reported by Medic-Alert subscribers.

#### Population Density

In this study we used the definition provided by Population Centre and Rural Area Classification 2016 of Statistics Canada.^1^ The categories used for this variable were urban and rural. This was a derived variable. Population density data were derived from the postal code. An area with a population of at least 1,000 and a population density of 400 persons or more per square kilometer was classified as an urban area; otherwise, it was classified as a rural area.

#### Living arrangement

This variable refers to whether or not the Medic-Alert subscriber lives with another person at home or in an institution. This variable was combined into four broader categories, i.e., with family, alone, institution, and other (see notes e and f on Table 1 for more details). Living arrangement data were self-reported by Medic-Alert subscribers.

#### Dementia status

In this study dementia refers as an umbrella term that describes a set of symptoms affecting brain function. Categories used for this variable were present and not present. Data on dementia status was self-reported by Medic-Alert subscribers.

### Operationalization of variables

Table 2 in supplemental material shows the operationalization used in the statistical model. The coding of the variables was completed by the authors. Dichotomous variables were coded as ‘‘0’’ or ‘‘1’’ (e.g., Medic-Alert subscriber’s sex at birth) and each polytomous variable (e.g., Medic-Alert subscriber’s ethnic background) was represented by a set of binary codes whose values codified each category variables. Concerning the outcome measure of interest in this study, namely a history of critical wandering, we dichotomized it as follows: a history of critical wandering coded as "never" was assigned a code of "0" (i.e., No); a history of critical wandering coded as "1–4 times" or "more than 4 times" was assigned a code of "1" (i.e., Yes).

**Table 2:** Multivariate logistics regression model of factors associated with wandering in Medic-Alert® subscribers (n = 25,785)

Variables ^a^	β	SE	Wald (*df*)	*p*-value	Odds ratio (95% CI)
**Age group **^**b**^					
≤ 65	.	.	.	.	1 (Reference)
65–74	1.04	0.07	217.77(1)	< 0.001	2.85 (2.48,3.27)
75–84	1.63	0.07	619.02(1)	< 0.001	5.10 (4.49, 5.80)
85–94	1.83	0.07	765.83(1)	< 0.001	6.27 (5.50, 7.14)
95–104	1.95	0.09	483.72.14(1)	< 0.001	7.11(5.97, 8.47)
≥ 105	2.67	0.54	24.56(1)	< 0.001	14.52 (5.04, 41.896)
**Sex at birth **^**b**^					
Female	.	.	.	.	1 (Reference)
Male	0.07	0.03	6.13 (1)	0.013	1.08 (1.02,1.15)
**Ethnic background **^**b**^					
Black	.	.	.	.	1 (Reference)
Chinese	-0.59	0.14	18.08(1)	< 0.001	0.55 (0.42,0.73)
Other	-0.28	0.11	6.18(1)	0.013	0.76 (0.61,0.94)
White (Caucasian)	-0.76	0.10	58.20(1)	< 0.001	0.47 (0.39, 0.57)
**Canadian languages spoken **^**b**^					
No	.	.	.	.	1 (Reference)
Yes	-0.54	0.08	43.97(1)	< 0.001	0.586 (0.50, 0.69)
**Population Density **^**b**^					
Rural	.	.	.	.	1 (Reference)
Urban	0.27	0.06	22.52 (1)	< 0.001	1.30 (1.17, 1.45)
**Living arrangement **^**b**^					
Alone	.	.	.	.	1 (Reference)
Institution	0.69	0.05	176.47(1)	< 0.001	1.99 (1.78,2.20)
With Family	0.21	0.04	23.78(1)	< 0.001	1.23 (1.13, 1.34)
Other	-0.09	0.10	0.92(1)	0.337	0.91 (0.75, 1.10)
**Dementia status **^**c**^					
Not present	.	.	.	.	1 (Reference)
Present	0.92	0.03	727.08 (1)	< 0.001	2.52 (2.35, 2.69)
**Constant**					
Constant	-2.13	0.156	199.221(1)	< 0.001	0.12 (0.16,0.27)
**Model**					
R^2^ (Nagelkerke)	0.230				
Correct classification of cases	71.5%				

The dependent variable in this analysis is critical wandering coded so that 0 = no wandering and 1 = wandering

^a^Variable domains/factors: Demographic: Age group, Ethnic background, Sex at birth, Canadian languages spoken. Environmental: Living arrangement, Population Density. Psychopathological: Dementia status

^b^Control variable

Independent variable

### Data sources and data collection procedures

The data are sourced from the Medic-Alert Canadian subscriber database. Medic-Alert utilizes three channels for data collection: [1] phone-survey intake, where the Medic-Alert subscriber verbally communicates responses during a phone call, and a call line operator enters the information into the system; [2] online intake via the Medic-Alert website; or [3] paper-based survey intake, where the Medic-Alert survey was sent to the subscriber, who completed the requested information. Regardless of the channel, Medic-Alert ensured that the collected information is of high quality. Specifically, a Medic-Alert profile specialist verified that critical information is completed and accurate, focusing on health-related details crucial for aiding the subscriber in an emergency scenario, such as a critical wandering incident. While the Medic-Alert subscriber was primarily responsible for completing the survey intake, there were instances where a care partner, authorized to act on behalf of the Medic-Alert subscriber, assisted in providing the required information, particularly in cases of cognitive impairment like dementia. In summary, the information provided by Medic-Alert subscribers was self-reported, however, every application underwent screening by a Medic-Alert profile specialist to verify the quality of the reported data from all Medic-Alert subscribers. Regarding the critical wandering variable, this information was collected through a specific section in the survey intake. Medic-Alert ensured that subscribers' information was updated at least annually, prompting them each year to review and revise any updated details. Therefore, our study utilized the most recent self-reported information regarding a history of critical wandering and for all data from Medic-Alert subscribers. In cases where updated information about a history of critical wandering was not available, we used the initial intake data of a history of critical wandering in the dataset.

### Statistical methods

We described the demographic, psychopathological, and situational factors of the Medic-Alert subscribers, by reporting the frequency and proportion for categorical variables and the mean (SD) for continuous variables. We also described the variables of interest by our outcome variable. For all variables, we combined categories into broader categories, when possible, to maximize the power of the logistic regression model [19]. We handled missing data by using multiple imputation to compute parameter estimates. We created five imputations using our 10 variables, including the dependent variable. We used χ^2^ test or Fisher’s exact test as appropriate to assess the differences between our variables of interest and the outcome variable. We used Principal Component Analysis with varimax rotation to reduce the dimensionality of the variables. The eigenvalue was set to a cut-off value of 1.0. We conducted the Kaiser–Meyer–Olkin measure of sampling adequacy and the Bartlett's test of sphericity to assess the adequacy of our data prior to the application of the Principal Component Analysis [20]. In addition, we calculated the determinant of the correlation matrix to assess multicollinearity of the variables [20]. Then, we used a multivariable logistic regression analysis of the outcome variable, including dementia status as psychopathological independent variable, controlling for the other demographic, and environmental and situational factors of interest in the model. We evaluated the performance of the logistic regression model with the receiver operating characteristic curve analysis and test characteristics [21]. Due to the cross-sectional design of our study, we did not estimate risk factors. Instead we estimated the association between demographic, psychopathological, and environmental factors and a history of critical wandering among Medic-Alert subscribers, both with and without dementia. All analyses were done with SPSS statistical analysis software v. 28.0. We used a p-value of 0.05 as the threshold for statistical significance.

## Results

### Characteristics of the sample

Table 1 presents the demographic, psychopathological, and environmental variables, as well as the prevalence of a history of critical wandering incidents versus no history of such incidents among Medic-Alert subscribers. The sample was comprised of mainly older adults, with 77.4% over the age of 65 (mean 75.42 SD = 14.34). The sample had slightly more female (54.2%) and were predominantly Caucasian (88.2%). In 96.6% of cases, they spoke at least one of the Canadian official languages (English or French). The bulk of the sample came from Ontario (61.6%) with the smallest numbers from the Yukon (0.07%) and Nunavut and Northwest Territories (0.042). The majority (89.9%) lived in urban areas. Most reported living with a family member (65.1%). A dementia diagnosis including Alzheimer’s disease or other dementias was present in 50.7% of the sample. Table 1 also shows that overall, the prevalence of a history of critical wandering was 28.5%, representing 7,349 unique individuals in the study period. As expected, the prevalence of a history of critical wandering were higher among older age groups, with the peak prevalence between ages 85–94 years. Although the difference in proportions of sex at birth were statistically significant, the absolute differences in a history of critical wandering for males and females were relatively modest (52.3%) and 47.7%, respectively, Δ_age_ = 4.6%). Of the 7,349 individuals with a history of critical wandering, 5,561 (75.7%) were presently living with a form of dementia or cognitive impairment.

## Multivariable Analysis

Figure A.1 and Tables A.3 and A.4 in Supplemental Material show the screen plot inspection eigenvalues and the items and their loadings on the factors of the Principal Component Analysis. This analysis suggests the existence of four factors that explain involvement in a history of critical wandering incidents account for 60% of the variance. The Kaiser–Meyer–Olkin measure of sampling adequacy (KMO = 0.608) and the Bartlett's test of sphericity (χ^2^[36] = 82,926.37, *p* < 0.000) indicates that our data were appropriate for the use of Principal Component Analysis for dimensionality reduction [20]. The determinant value of our correlation matrix was 0.528, which was greater than the cut-off value of 10^–5^ [20], indicating multicollinearity was not an issue in this dataset. The province/territory variable was not included in the final multivariable logistic regression analysis as it showed similarities with the population density variable.

**Fig.1:**
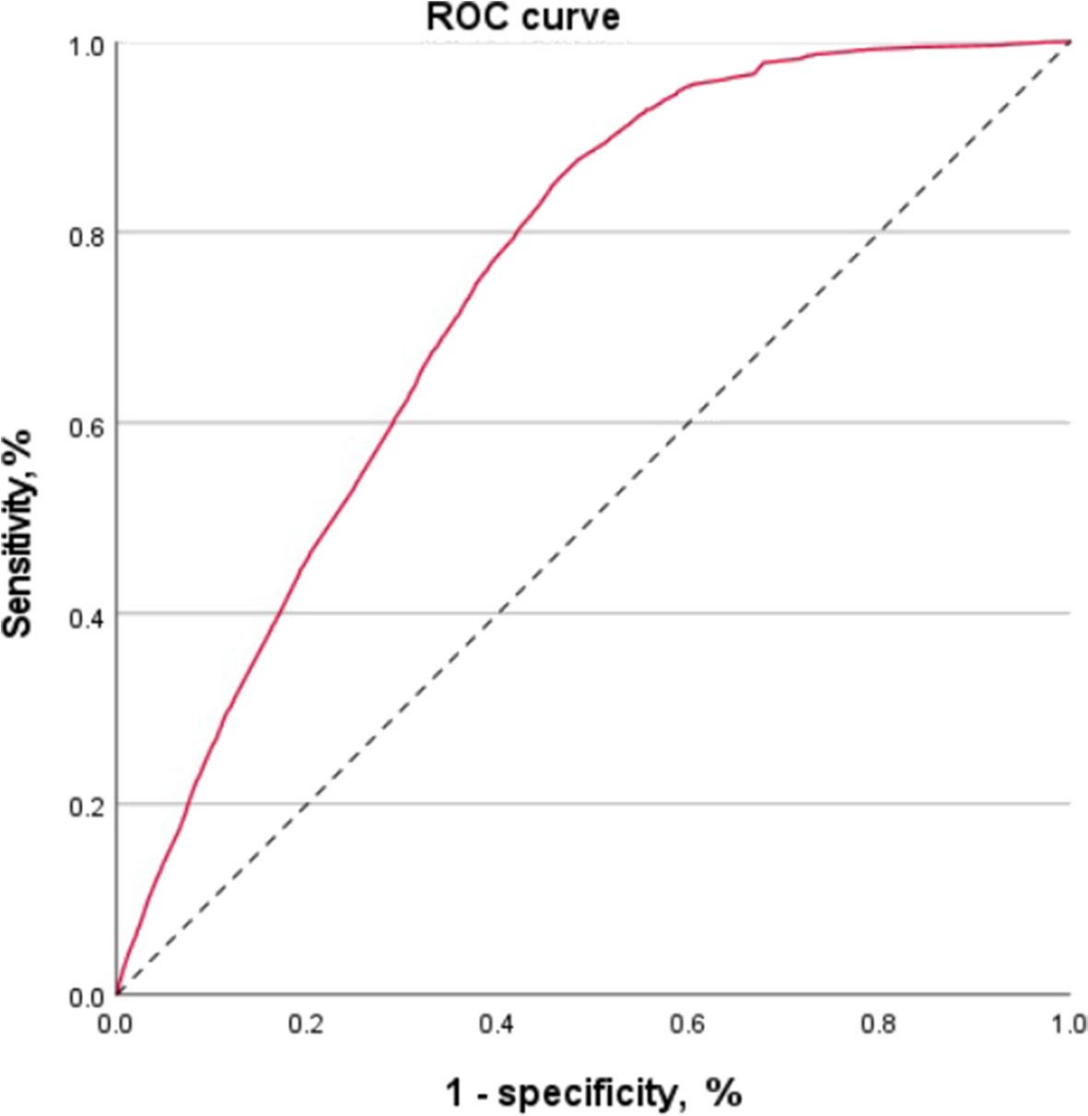
Receiver Operating Characteristic Curve. A wandering score of 1 indicates Medic-Alert subscribers wandered at least one (i.e., has a history of critical wandering)

Table 2 shows the results of our multivariate logistics regression analysis. We found that Medic-Alert subscribers who were older, male at birth, had a black ethnic background, were not proficient in a Canadian language, lived in an urban environment, in an institution or with a family member, and a dementia diagnosis were associated with higher likelihood of an outcome of a history of critical wandering. Notably for the predictor variable of dementia status, the odds in favour of having a history of critical wandering was 2.52 (95% CI, [2.35–2.69], *p*-value < 0.001) times larger for a person who has dementia than a person without dementia.

Figure 1 shows the receiver operating characteristic curve of the model. The area under the curve was 0.741 (95% CI, 0.738–0.750, *p*-value = 0.000), indicating a fair ability of the model to classify Medic-Alert subscribers with a history of critical wandering behaviors for from those who do not. Also, we obtained the classifying power of the model being 71.50%, meaning that the model has a good power of classification. The Nagelkerke Pseudo R^2^ obtained was 0.230 indicated a large effect size [22].

## Discussion

This study aimed to explore the association between demographic, psychopathological, and environmental factors of a history of critical wandering among individuals living with and without dementia. To our knowledge, this is the first study to address a major shortcoming in the existing literature on this topic; that is, small sample sizes, poor data quality, and missing data. The sample of 25,785 unique Medic-Alert Foundation Canada subscribers examined in our study notably surpasses what is typical of this literature [23]. We found the likelihood of a history of critical wandering was higher in people who were age 65 and older, live with dementia, are visible minorities (specifically those who have a black ethnic background), and are not proficient in a Canadian language.

The finding that older people living with dementia are at increased likelihood of critical wandering did not surprise us. It is evident that the combined effect of normative age-related declines in perceptual, cognitive, and psychomotor performance [24] with medical conditions such as Alzheimer’s or other dementias explain the increased risk [16]. The neurocognitive deficits such as remembering facts and events, short term memory for recent events and contexts, or lack of insight, executive function impairments, and difficulty with navigation that occur as dementia progresses predisposes a person living with dementia to critical wandering and getting lost [16].

Our analyses revealed that people from visible minorities, specifically those from a black ethnic group, were associated with higher risk of a history of critical wandering. This discrepancy in the risk of a history of critical wandering among ethnic groups can be partially explained by the higher risk of developing dementia in this population [25]. Higher rates of diabetes and hypertension in this population are known precursors to dementia and have been attributed to chronic stressors associated with race-based discrimination [26].

Critical wandering and the higher risk of getting lost among visible minority individuals living with dementia can be attributed to several reasons. First, our study found that those who are not proficient in one of the two Canadian official languages (English or French) were associated with higher risk for a history of critical wandering or getting lost (e.g., we found that in 45.5% of cases Medic Alert® subscribers with a Chinese ethnic background did not speak one of the two Canadian official languages, see Table A.5, Supplemental Material). This result is similar to other studies where language barriers have been associated with increased risk of negative health outcomes, such as hospital admission, increased risk of intubation, differences in prescribed medication, greater number of reported adverse drug reactions, and lower rates of optimal pain medication [27]. Second, studies have reported a notable delay in the diagnosis of dementia among Black individuals due to low education levels of caregivers, cultural differences, and racial biases in the healthcare system [28]. Cultural differences may include the incorrect assumption that dementia is an inevitable part of aging and can lead to apprehension towards treatment [29]. These delays in diagnoses consequently result in delayed treatment, less opportunity for care, and more adverse outcomes like critical wandering [30]. All socioecological levels, including individual language and ethnicity, caregiver support, community resources and even cultural norms, may explain this disproportionate risk of critical wandering among ethnic groups, specifically Black Canadians. Finally, research has shown that experiences of discrimination, racial profiling, and mistreatment can create deep-seated distrust and fear of authorities within minority communities, including law enforcement [31]. This fear may discourage individuals or their families from seeking help when someone goes missing, which can delay search and rescue efforts, increase the risk of harm, and undermine public safety. Consequently, building trust between minority communities and law enforcement is crucial to addressing the elevated risk of critical wandering and reducing potential harm to this vulnerable population. We also found that Medic-Alert subscribers of Chinese ethnic background constituted another visible minority facing an elevated risk of a history of critical wandering. In addition to their lack of proficiency in one of the two Canadian official languages, the heightened risk of a history of critical wandering within this group can be attributed to two factors. First, individuals from the Chinese ethnic background often experience acculturation stress due to the challenges of adapting to a new culture, potentially resulting in cognitive and behavioral challenges [32], including an increased risk of critical wandering. Second, studies have reported that cultural attitudes toward seeking medical help, influenced by stigma surrounding dementia, could contribute to the observed differences [33]. It is well-documented that Chinese-Americans associate dementia with stigma and a "loss of face" [34]. The concept of “loss of face” is deeply embedded in East Asian cultures, including Chinese culture, referring to the potential embarrassment, shame, or loss of dignity that an individual or their family may experience in the eyes of others. In the context of dementia, this fear becomes particularly pronounced due to the cognitive and behavioral changes associated with the condition. Thus, the fear of losing face may deter Chinese-American families from seeking help when they are involved in a missing incident due to critical wandering, stemming from concerns about external judgment and the perceived failure to fulfill cultural expectations.

Our finding suggesting that a history of critical wandering is associated with a higher risk of getting lost among visible minority individuals living with dementia should be interpreted with caution. The MedicAlert dataset, comprising approximately 11.8% visible minorities (1.9% black individuals), was not representative of the Canadian population which is 26.5% visible minority and 4.3% black^2^. Clearly, barriers such as financial constraints, cultural differences, or lack of awareness may hinder people from visible minorities in becoming MedicAlert subscribers. Possibly, among these ethnic groups, individuals with a history of critical wandering events were more inclined to be enrolled in the MedicAlert dataset. In summary, due to the dataset's lack of representativeness, it is likely our results showed a bias towards visible minorities who had experienced critical wandering events.

Another finding was that those who lived with family or in an institution were associated with higher history of critical wandering prevalence. We believe this may not a risk factor in itself, but a confounder. It is estimated that care partners spend 26 h per week on average caring for persons living with dementia [35], with tasks such as personal care (e.g., bathing, feeding) as well as keeping persons living with dementia safe in their homes by providing supervision [16, 36]. In several cases, caregiving tasks are fulfilled by a single individual, leaving the person living with dementia temporarily unsupervised while they complete their own personal hygiene, sleep, and fulfill household responsibilities [37]. In our opinion, temporary unsupervised situation is the true risk factor that leads to a higher risk of critical wandering or a missing incident. Due to the limitations of the dataset, we are unable to determine if a person living with dementia was left unsupervised preceding the critical wandering incident.

### Recommendations for Future Research and Practice

The results of this study have implications for future research and practice. First, the dataset used in this study represents a small portion of people living with dementia in Canada; because it is a paid subscription service, not everyone uses it. In future research, other sources of data such as police or search and rescue data should be included for a more representative data source. Second, before our risk model is adopted in actual practice, further research is needed for external validation, assessment of accuracy in a community setting, and determination of its utility for search and rescue tasks. Third, algorithms using data from organizations such as Medic-Alert could be used to flag risk of critical wandering among subscribers. This can inform preventative strategies at the individual level and can be combined with geospatial information to enable first responders to understand risk factors of members in the database. Fourth, our study demonstrated that in addition to dementia, multiple factors affect persons with wandering behaviours. This has implications for care partners, first responders and search and rescue personnel, the general public (i.e., Good Samaritans), and the healthcare system in instances where people living with dementia become lost. For example, search and rescue members can receive training on approaches to help people with dementia before, during and after a missing incident. Finally, future work to develop a comprehensive assessment tool for persons who are at risk of getting lost due critical wandering could consider individual-level risk factors along with environmental and situational domains.

### Study Limitations

This study has limitations. First, the cross-sectional nature of the dataset used in this study limits our ability to examine causal effects. It is evident that the experience of having multiple or repeated critical wandering episodes increases with dementia progression. We were not able to capture the dynamic nature of dementia progression and the correlational changes with frequency of wandering behaviours. Second, our source of data was a limitation as critical wandering behaviours were captured in a subscription-based registry. Therefore, the data were subjected to inherent self-selection biases. Third, information recorded in the dataset was self-reported instead of being provided or confirmed by health providers. That is, a Medic-Alert subscriber or care partners were at liberty to disclose or not disclose details about the subscriber’s diagnoses. As a result, true prevalence of dementia in this sample may be underestimated. Fourth, dementia subtypes were not available; consequently, we were unable to compare and estimate the effects of dementia subtypes on the risk of critical wandering.

### Generalisability

As the MedicAlert dataset did not represent visible minorities in Canada, this poses challenges to the external validity of the study results. In the dataset, overrepresentation of visible minorities who experienced critical wandering events may lead to an overestimation of the association between critical wandering and the risk of getting lost among the visible minority groups. Consequently, the study results may not accurately reflect the experiences and outcomes of individuals from visible minority groups who did not exhibit critical wandering behavior or were not enrolled in MedicAlert. Therefore, while the study provides valuable insights into the association between critical wandering and the risk of getting lost among individuals living with dementia, caution is warranted when extrapolating these findings to the broader population.

Despite these limitations, the results contribute to an understanding of factors associated with the phenomenon of critical wandering and missingness in older adults with dementia in Canada.

## Conclusions

In conclusion, there are multiple factors associate with wandering and lost behaviour. Our study shows that a history of critical wandering behaviour is a prevalent issue (i.e., 28.5%) among Medic-Alert Foundation Canada subscribers. It poses considerable risk to the person involving psychopathological, demographic and environmental and situational factors should they become lost. We developed a model to estimate the risk of going missing due to a history of critical wandering, stratifying the risk among individuals with and without dementia, with modest discrimination. These results can contribute to development of a model based on more representative data toward a risk assessment and management tool.

### Supplementary Information

**Supplementary Material 1.**

## Data Availability

The datasets generated and/or analyzed during the current study are not publicly available due to the restriction under the institutional ethical committee’s policy and they are part of a registry. Analytic methods are available (under request) to other researchers for replication purposes. For requests regarding datasets analyzed in the study please contact the corresponding author.

## Abbreviations

SD: Standard deviation
KMO: Kaiser-Meyer-Olkin measure of sampling adequacy
CI: Confidence interval
SPSS: Statistical Package for Social Sciences
SE: Standard error
Df: Degrees of freedom
ROC: Receiver Operating Characteristic Curve

## Footnotes

1 https://www.statcan.gc.ca/en/subjects/standard/pcrac/2016/introduction

2 https://www150.statcan.gc.ca/n1/daily-quotidien/221026/dq221026b-eng.htm
