# Supplementary material for: Factors associated with a history of critical wandering among Medic-Alert subscribers
Source: BMC Geriatr. 2024 Jun 28;24:564. doi: 10.1186/s12877-024-05162-3 (PMC11212194; doi:10.1186/s12877-024-05162-3)
Supplement: Supplementary file 1 — Supplementary Material 1. [file 12877_2024_5162_MOESM1_ESM.docx]

**Supplemental material**

Table A.1. Demographics, psychopathological and environmental characteristics before and after multiple imputation procedure (n= 25,785).

| **Variable** | **Before multiple imputation**  **n (%)** | **After multiple imputation**  **n (%)** |
| --- | --- | --- |
| **Age group** |  |  |
| <65 | 5,827 (22.6) | 5,827 (22.6) |
| 65-74 | 4,296 (16.7) | 4,296 (16.7) |
| 75-84 | 7,554 (29.3) | 7,554 (29.3) |
| 85-94 | 7,036 (27.3) | 7,036 (27.3) |
| 95-104 | 1,056 (4.1) | 1,056 (4.1) |
| ≥105 | 16 (0.1) | 16 (0.1) |
| Missing data | 0 (0) | 0 (0) |
| **Sex at birth** |  |  |
| Female | 13,688 (53.1) | 13,963 (54.2) |
| Male | 11,617 (45.2) | 11,822 (45.8) |
| Missing values | 480 (1.9) | 0 (0) |
| **Ethnic background** |  |  |
| White (Caucasian) | 22,127 (85.8) | 22,754 (88.2) |
| Other ^a^ | 2,569 (10) | 1,942 (7.5) |
| Chinese | 598 (2.3) | 598 (2.3) |
| Black ^b^ | 491 (1.9) | 491 (1.9) |
| Missing values | 627 (2.4) | 0 (0) |
| **Canadian spoken ^c^** |  |  |
| Yes | 24,915 (96.6) | 24,915 (96.6) |
| No | 870 (3.4) | 870 (3.4) |
| **Province/Territory** |  |  |
| Ontario | 11,933 (46.3) | 15,967 (61.9) |
| Quebec | 31,54 (12.2) | 3,154 (12.2) |
| British Columbia | 2,849 (11) | 2,849 (11.0) |
| Alberta | 1,570 (6.1) | 1,570 (6.1) |
| Manitoba | 815 (3.2) | 815 (3.2) |
| Nova Scotia | 550 (2.1) | 550 (2.1) |
| Saskatchewan | 398 (1.5) | 398 (1.5) |
| New Brunswick | 263 (1) | 263 (1.0) |
| Newfoundland and Labrador | 125 (0.5) | 125 (0.5) |
| Prince Edward Island | 65 (0.3) | 65 (0.3) |
| Yukon | 18 (0.1) | 18 (0.07) |
| Nunavut/Northwest Territories | 11 (0.0) | 11(0.042) |
| Missing values | 4,034 (15.6) | 0 (0) |
| **Population Density** |  |  |
| Urban | 19,583 (75.9) | 23,185 (89.9) |
| Rural | 2,161 (8.4) | 2,600 (10.1) |
| Missing values | 4,041 (15.7) | 0 (0) |
| **Living arrangement** |  |  |
| With Family | 16,798 (65.1) | 16,798 (65.1) |
| Alone | 4,513 (17.5) | 4,513 (17.5) |
| Institution | 3,368 (13.1) | 3,576 (13.9) |
| Other | 898 (3.5) | 898 (3.5) |
| Missing values | 208 (0.8) | 0 (0) |
| **Dementia status** |  |  |
| Present | 13,064 (50.7) | 13,064 (50.7) |
| Not present | 12,721 (49.3) | 12,721 (49.3) |
| Missing values | 0 (0) | 0 (0) |
| Notes   1. Others (Arab/West Asian (e.g., Armenian, Egyptian, Iranian), Latin American, South Asian, Korean, Mediterranean, first nations (e.g., Inuit, Métis, North American Indian), Filipino, Caribbean/West Indian (Lucia, Antigua), Southeast Asian, and Japanese 2. African. Haitian. Jamaican. Somali 3. English or French | | |

Table A.2 Variables operationalization (included in the logistic regression model)

| **Variables** | **Domain** | **Categories** | **Variable operationalization** | | | | |
| --- | --- | --- | --- | --- | --- | --- | --- |
|  |  |  | **(1)** | **(2)** | **(3)** | **(4)** | **(5)** |
| Age group | Demographic | <65 | 0.0 | 0.0 | 0.0 | 0.0 | 0.0 |
|  |  | 65-74 | 1.0 | 0.0 | 0.0 | 0.0 | 0.0 |
|  |  | 75-84 | 0.0 | 1.0 | 0.0 | 0.0 | 0.0 |
|  |  | 85-94 | 0.0 | 0.0 | 1.0 | 0.0 | 0.0 |
|  |  | 95-104 | 0.0 | 0.0 | 0.0 | 1.0 | 0.0 |
|  |  | >105 | 0.0 | 0.0 | 0.0 | 0.0 | 1.0 |
| Living arrangement | Environmental and situational | Alone | 0.0 | 0.0 | 0.0 |  |  |
|  |  | Institution | 1.0 | 0.0 | 0.0 |  |  |
|  |  | With Family | 0.0 | 1.0 | 0.0 |  |  |
|  |  | Other | 0.0 | 0.0 | 1.0 |  |  |
| Ethnic background | Demographic | Black | 0.0 | 0.0 | 0.0 |  |  |
|  |  | Chinese | 1.0 | 0.0 | 0.0 |  |  |
|  |  | Other | 0.0 | 1.0 | 0.0 |  |  |
|  |  | White (Caucasian) | 0.0 | 0.0 | 1.0 |  |  |
| Sex at birth | Demographic | Female | 0.0 |  |  |  |  |
|  |  | Male | 1.0 |  |  |  |  |
| Canadian languages spoken | Demographic | No | 0.0 |  |  |  |  |
|  |  | Yes | 1.0 |  |  |  |  |
| Population Density | Environmental and situational | Rural | 0.0 |  |  |  |  |
|  |  | Urban | 1.0 |  |  |  |  |
|  |  | Yes | 1.0 |  |  |  |  |
| Dementia status | Psychopathological | No | 0.0 |  |  |  |  |
|  |  | Yes | 1.0 |  |  |  |  |

Figure A.1. Scree plot.


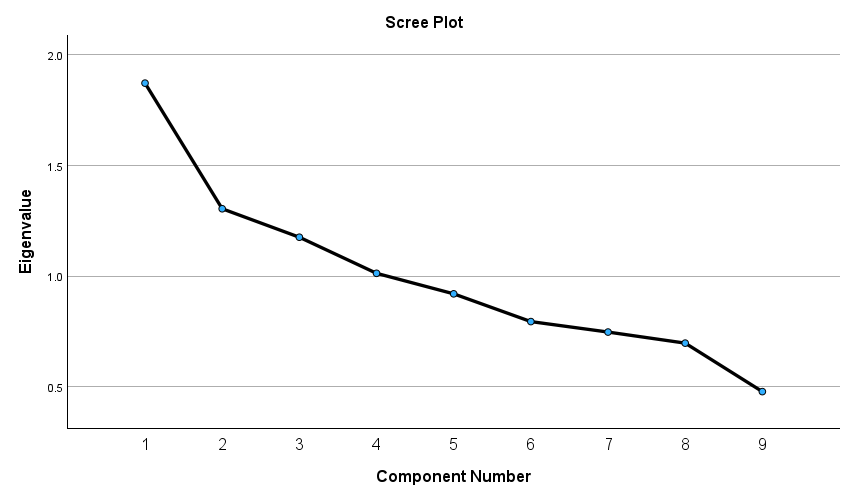


Table A.3. Total variance explained.

| Component | Initial eigenvalues | | | Extraction sums of squared loadings | | | Rotation sums of sums of squared loadings | | |
| --- | --- | --- | --- | --- | --- | --- | --- | --- | --- |
|  | Total | % of variance | Cumulative % | Total | % of variance | Cumulative % | Total | % of variance | Cumulative % |
| 1 | 1.872 | 20.798 | 20.798 | 1.872 | 20.798 | 20.798 | 1.795 | 19.943 | 19.943 |
| 2 | 1.304 | 14.491 | 35.289 | 1.304 | 14.491 | 35.289 | 1.349 | 14.985 | 34.928 |
| 3 | 1.175 | 13.061 | 48.350 | 1.175 | 13.061 | 48.350 | 1.176 | 13.062 | 47.990 |
| 4 | 1.012 | 11.250 | 59.600 | 1.012 | 11.250 | 59.600 | 1.045 | 11.610 | 59.600 |
| 5 | 0.920 | 10.219 | 69.820 |  |  |  |  |  |  |
| 6 | 0.794 | 8.826 | 78.646 |  |  |  |  |  |  |
| 7 | 0.747 | 8.300 | 86.946 |  |  |  |  |  |  |
| 8 | 0.697 | 7.744 | 94.690 |  |  |  |  |  |  |
| 9 | 0.478 | 5.310 | 100.000 |  |  |  |  |  |  |

Table A.4. Items and their loadings on the four factors

| **Rotated component matrix ^a^** | | | | |
| --- | --- | --- | --- | --- |
|  | Component | | | |
|  | 1 | 2 | 3 | 4 |
| Age group | 0.812 | 0.017 | -0.098 | -0.034 |
| Sex at birth | 0.159 | 0.144 | 0.791 | -0.111 |
| Ethnic background | 0.006 | 0.790 | 0.004 | 0.009 |
| Canadian languages spoken | -0.128 | 0.724 | -0.010 | -0.004 |
| Province/Territory | 0.081 | -0.239 | 0.000 | 0.745 |
| Population Density | 0.117 | -0.285 | -0.039 | -0.666 |
| Living arrangement | -0.202 | -0.161 | 0.733 | 0.164 |
| Dementia status | 0.798 | -0.045 | 0.025 | 0.054 |
| Wandering history | 0.629 | -0.116 | 0.033 | -0.054 |
| Extraction methos: PCA.   Rotation method: Varimax with Kaiser normalization.  a. Rotation converged in 5 iterations. | | | | |

Table A.5. Cross table: Ethnic background Canadian languages spoken

| **Ethnic background** | **Canadian languages spoken** | | | | | |
| --- | --- | --- | --- | --- | --- | --- |
|  | **No** | | **Yes** | | **Total** | |
|  | **n** | **%** | **n** | **%** | **n** | **%** |
| Black | 11 | 2.2 | 480 | 97.8 | 491 | 100 |
| Chinese | 272 | 45.5 | 326 | 54.5 | 598 | 100 |
| Other | 260 | 13.4 | 1,682 | 86.6 | 1,942 | 100 |
| White (Caucasian) | 327 | 1.4 | 22427 | 98.6 | 22,754 | 100 |
| Total | 870 | 3.4 | 24,915 | 96.6 | 25,785 | 100 |
